# Sensitivity analysis of coupled processes and parameters on the performance of enhanced geothermal systems

**DOI:** 10.1038/s41598-017-14273-4

**Published:** 2017-12-06

**Authors:** S. N. Pandey, Vikram Vishal

**Affiliations:** Computational and Experimental Geomechanics Laboratory, Department of Earth Sciences, Indian Institute of Technology Bombay, Mumbai, 400076 India

## Abstract

3-D modeling of coupled thermo-hydro-mechanical (THM) processes in enhanced geothermal systems using the control volume finite element code was done. In a first, a comparative analysis on the effects of coupled processes, operational parameters and reservoir parameters on heat extraction was conducted. We found that significant temperature drop and fluid overpressure occurred inside the reservoirs/fracture that affected the transport behavior of the fracture. The spatio-temporal variations of fracture aperture greatly impacted the thermal drawdown and consequently the net energy output. The results showed that maximum aperture evolution occurred near the injection zone instead of the production zone. Opening of the fracture reduced the injection pressure required to circulate a fixed mass of water. The thermal breakthrough and heat extraction strongly depend on the injection mass flow rate, well distances, reservoir permeability and geothermal gradients. High permeability caused higher water loss, leading to reduced heat extraction. From the results of TH vs THM process simulations, we conclude that appropriate coupling is vital and can impact the estimates of net heat extraction. This study can help in identifying the critical operational parameters, and process optimization for enhanced energy extraction from a geothermal system.

## Introduction

Geothermal energy is clean, renewable, sustainable and has the potential of providing base load energy. The major advantage of geothermal energy over other sources of renewable energy is that the underground heat/energy mining does not depend on the weather conditions. In the past six years (2010–2015) the direct utilization of geothermal energy has increased by 31.58% reaching upto 70,885 MWt worldwide^1^. In addition to direct use, the electricity generation installed capacity has also reached to 12.729 GW in 2015 from 10.895 GW in 2010 with a growth of 14.40%^2^. However, geothermal energy has less than 0.5% share of current electricity generation. By 2050, geothermal energy could be able to provide approximately 3.5% of the world energy demand^3^.

In an EGS (enhanced geothermal systems) reservoir, heat is extracted by injecting cold water into the fractures and pumping out hot water after receiving the heat from the reservoir. Fractures/joints in EGS reservoir play a significant role because they are the main flow conducts and have higher transmissivity compared to the surrounding host rock matrix. The fracture in reservoir acts as a heat exchanger and the heat extraction performance of the reservoir depends on the volume of fracture/reservoir participating in the flow process. Flow field evolution due to change in reservoir temperature and stresses is an important feature of the EGS but not necessarily accounted for in several studies^4–16^. The cooling and pore pressure changes have contrasting effects on the reservoir. While cooling causes contraction, increase in pore pressure causes expansion of the reservoir matrix and collectively result in the evolution of fracture porosity/permeability^17–26^. The spatial-temporal variation of fracture aperture during injection/production significantly influences the heat extraction from the reservoir^22–25^. These studies have indicated that coupled thermo-hydro-chemical-mechanical processes and their effects on heat extraction are present throughout the reservoir operation. The pore pressure effects are important during early stages while the thermal and chemical effects become increasingly dominant during intermediate to long durations of injection/production operation. However, over time, the combined effects result in either loss or gain of injectivity depending on the relative magnitude of these processes, reservoir mineralogy and operational conditions. These coupled effects should therefore be understood in great details for long term, sustainable utilization of an EGS reservoir.

A fully coupled thermo-hydro-mechanical model for field scale geothermal system that combines physical processes as well as operating parameters is rarely found in literature. The recent modeling study of Guo *et al*.^24^, Pandey *et al*.^25^ have focused on reservoir parameters such as joint stiffness, heterogeneity, and coefficient of thermal expansion on aperture evolution and heat extraction in a THM framework. They found that the opening of a fracture between the injection and production wells is the main cause for flow channeling and fast temperature drop at the production well. The effects of heterogeneities were prominent when the correlation lengths were larger. For small correlation lengths, the flow behavior is almost same as that for a uniform fracture. Rawal and Ghassemi^22^ studied the effects of injection concentration and joint stiffness in a THMC (thermo-hydro-mechanical-chemical) framework. They showed that the aperture evolution between the wells is primarily controlled by the THM effects. The chemical effects became almost negligible due to the reduction of reaction kinetics of quartz due to cooling. They also showed that a higher joint stiffness provides more resistance that results in slower aperture growth. Few studies have considered the geochemical effects due to dissolution/precipitation of reservoir minerals^27–30^. Such geochemical reactions become significant when the reservoir lies in a carbonate or sandstone formation^30^. Since most of the EGS reservoirs lie in granite formation, the aperture evolution due to water-rock reaction is negligible within the typical lifespan (~30 years) of the reservoir^31,32^.

Along with reservoir evolution due to the THMC processes, the heat extraction performance is also affected by the operational parameters. Such parameters include injection temperature, mass flow rate, injection fluid properties, well distance, reservoir thermal gradient and fractures spacing and the fracture density.

The overall performance of an EGS is controlled by its rock-mechanical parameters together with the evaluation of transport properties. In this paper, a fully coupled thermo-hydro-mechanical (THM) modeling of cold water injection into the geothermal reservoir was carried out using a 3-D enhanced geothermal system. This model was formulated by considering all vital aspects, including the reservoir formation properties and operational parameters of an enhanced geothermal system. The fracture was modeled as an equivalent porous medium and implemented in FEHM (Finite Element Heat and Mass Transfer) code. The nonlinear stress dependent fracture joint model was used for mechanical deformation of fracture. The pressure and temperature dependence of fluid and mechanical properties of the rock were considered. The study is novel in two aspects: (i) focus on coupling effects on energy production, and (ii) sensitivity analysis of key parameters. The results of this study provide valuable information for enhanced geothermal system during operational period under varying reservoir conditions. The approach/methodology can be suitably used and applied to other subsurface systems (transport of CO_2_, oil, water, etc. in different geological reservoirs) and their geomechanical responses to the flow investigated.

## Mathematical Model

Flow and heat transport are described separately for fracture and matrix in the reservoir using a set of partial differential equations. For modeling of THM processes, key governing equations for the conservation of mass, momentum, energy and mechanical balance equations are described below:

The mass and momentum balance for fluid flow in a fracture can be represented using quasi-steady state equations^33–35^:

1 $$\nabla .{{\bf{Q}}}_{f}={f}_{Q}\,{\rm{and}}\,{{\bf{Q}}}_{f}=-\frac{{b}^{3}}{12\mu }(\nabla {P}_{f}-\rho {\bf{g}})$$

where ${{\bf{Q}}}_{f},b,{P}_{f},\mu ,\rho ,{\bf{g}}$ and ${f}_{Q}$ are the aperture-integrated two-dimensional flux vector, fracture aperture, aperture-averaged pressure, dynamic viscosity, the density of the water, gravitational acceleration and lateral exchange of fluid between the fracture and permeable rock, respectively. The flow in the low permeability porous matrix is modeled by mass balance equation and Darcy law:

2 $$\nabla \cdot {{\bf{q}}}_{r}=0\,{\rm{and}}\,{{\bf{q}}}_{r}=-\frac{k}{\mu }(\nabla {P}_{r}-\rho {\bf{g}})$$

where $k,\,{{\bf{q}}}_{r}$ and ${P}_{r}$ are the permeability, Darcy flux and fluid pressure in porous rock matrix, respectively. The coupling between flow through fracture and rock matrix is incorporated using the following relations:

3 $${f}_{Q}={{q}_{rz}|}_{z=b/2}-{{q}_{rz}|}_{z=-b/2}\,{\rm{and}}\,{{P}_{r}|}_{z=-b/2}={{P}_{r}|}_{z=b/2}={P}_{f}$$

Here $z=-b/2$ and $z=b/2$ represent the upper and lower surfaces of the fracture within the rock matrix. The second component in Eq. (3) corresponds to pressure continuity.

The energy balance equations for 2-D fracture and 3-D reservoir matrix are:

4 $$\frac{\partial (b{(\rho {c}_{p})}_{f}{T}_{f})}{\partial t}+{{\bf{Q}}}_{f}\cdot \nabla {h}_{f}-b\nabla \cdot ({\lambda }_{f}\nabla {T}_{f})={f}_{T}$$

5 $${\rm{and}}\,\frac{\partial ({(\rho {c}_{p})}_{r}{T}_{r})}{\partial t}+{{\bf{q}}}_{r}\cdot \nabla {h}_{r}-\nabla \cdot ({\lambda }_{r}\nabla {T}_{r})=0$$

where $T,\,\lambda ,\,h$ and ${c}_{p}$ are the temperature, thermal conductivity, enthalpy of water and specific heat, respectively. The subscripts *f* and $r$ are used to represent the fracture and the rock. The heat capacity of the rock matrix is defined as ${(\rho {c}_{p})}_{r}=(1-\varphi ){(\rho {c}_{p})}_{{\rm{solid}}}+\varphi {(\rho {c}_{p})}_{{\rm{fluid}}}$. Similar to the flow equations, the heat transport equations are coupled through the heat flux $({f}_{T})$ at the fracture and matrix interfaces. The temperature continuity at $z=\pm b/2$ is:

6 $${f}_{T}={({q}_{rz}{h}_{r}-{\lambda }_{r}\frac{\partial {T}_{r}}{\partial z})}_{z=b/2}-{({q}_{rz}{h}_{r}-{\lambda }_{r}\frac{\partial {T}_{r}}{\partial z})}_{z=-b/2}\,{\rm{and}}\,{{T}_{r}|}_{z=-b/2}={{T}_{r}|}_{z=b/2}={T}_{f}$$

The force balance equation for the fluid–rock assuming quasi-steady state is given as:

7 $$\nabla \cdot {\boldsymbol{\sigma }}+{\rho }_{r}{\bf{g}}=0$$

where ${\bf{g}}$ is the vector of body forces and ${\rho }_{r}$ is the rock density.

The governing equation for thermo-poro-elastic processes can be expressed as:

8 $$\sigma =\lambda tr(\varepsilon )+2G\varepsilon +(\beta {\rm{\Delta }}{P}_{r}+K\alpha {\rm{\Delta }}{T}_{r}){\bf{I}}$$

where $\beta$, $\alpha$, ${\bf{I}}$ and $tr()$ are the Biot’s coefficient, linear thermal expansion coefficient, second order identity tensor and the trace operator, respectively.

The fracture opening and closing was modeled using nonlinear hyperbolic, Bandis and Barton model^36^. The relation between fracture aperture and effective normal stresses is given as:

9 $$b={b}_{{\rm{\max }}}-\frac{A{\sigma ^{\prime} }_{n}}{1+B{\sigma ^{\prime} }_{n}}$$

where ${\sigma ^{\prime} }_{n}$ is the effective stress, ${b}_{\max }$ is the maximum fracture opening at zero effective stress, *A* and *B* are the two controlling parameters that depend on mechanical properties of the joint rock. The stiffness of fracture joint is defined as:

10 $${K}_{n}=\frac{d{\sigma ^{\prime} }_{n}}{db}=\frac{{(1+B{\sigma ^{\prime} }_{n})}^{2}}{A}$$

Heat extraction from the geothermal reservoir for a pair of injection and production well is given by:

11 $$\dot{E}=\dot{m}({h}_{pro}-{h}_{inj})$$

where $\dot{m}$ is the mass flow rate of water at production well, ${h}_{inj}$ is the enthalpy of injected water and ${h}_{pro}$ is the enthalpy of water at the production well. The enthalpy of water is considered as a function of pressure and temperature, $h=f(P,T)$.

## Numerical modeling

**Figure 1:**
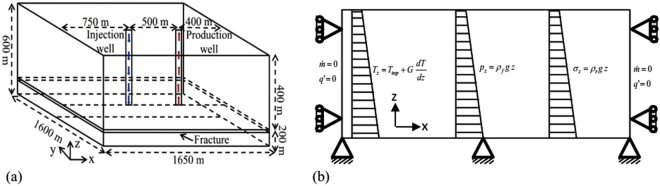
Schematic diagrams of (**a**) geothermal heat extraction system^25^ and (**b**) applied boundary conditions.

The coupled thermo-hydro-mechanical modeling of geothermal reservoir was performed using control volume finite element code, FEHM^37^. FEHM solves flow and heat transport equations using control volume method and deformation equation through finite element method. FEHM is a well validated code, validated against commercial simulators (Abaqus, CMG and STARS), analytical solutions and the field data of enhanced geothermal systems^38^. FEHM integrates all multi-physics processes in a single code and the effects of one process on the other are coupled through different methods based on the problem of interest, which helps to minimize the computational cost and time depending on the complexity. Detail description of solving the governing equations, coupling methods are explained by Kelkar *et al*.^38^. The fracture was modeled as an equivalent porous medium and the approach was validated for different thickness of porous layers in previous works^25^. In this study, simulations were conducted in a fracture-matrix system involving transport of fluid in a highly permeable horizontal fracture, connecting injection and production wells as shown in Fig. 1a. The reservoir is modeled as a three dimensional domain with a length of 1.65 km in the $x$ and 1.6 km in the $y$ and 600 m in the $z$ direction, respectively (Fig. 1a). The top surface of computational domain (reservoir) is located 1.6 km below the ground surface and the domain extends upto a depth of 2.2 km depth. The fracture in the reservoir was located at 2 km depth. The initial fracture aperture was considered 0.147 mm before water injection. The temperature of ground surface was assumed as 30 °C. Scenarios of varying geothermal gradients, 60, 80 and 100 °C/km were considered and used to define the initial temperature distributions inside the reservoir. Hence, the temperatures along the fracture surface were 150, 190 and 230 °C in each scenario before the fluid injection began. Fluid pressure (pore pressure) in the reservoir is initially hydrostatic and vertical gradient with depth is applied as an initial condition. The regional flow and tectonic activities were neglected. No flow boundary condition was imposed at all boundaries and zero heat flux (adiabatic) at the lateral boundaries i.e. excluding the top and bottom boundaries (constant temperature at top and bottom were used to define the geothermal gradient). The lithostatic stress follow the stress-depth relation. The bottom boundary was specified as zero displacement (fixed) and other surfaces were placed on roller. The schematic diagram of the applied boundary conditions in the model is given in Fig. 1b. Temperature and pressure dependent water properties such as density, viscosity, enthalpy etc. are inbuilt in the FEHM code. Thermal drawdown, heat extraction, and pressure required to circulate the fixed mass of water were shown through numerical simulations.

## Results and Discussion

**Figure 2:**
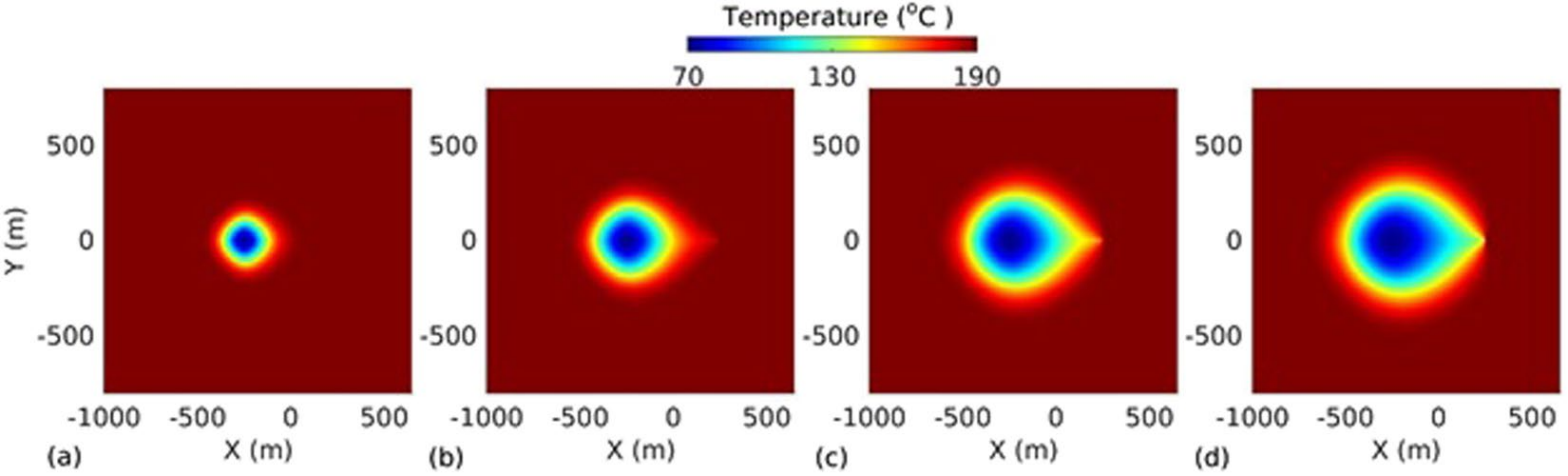
Temperature distribution on the fracture plane at different time instances: (**a**) 1 year, (**b**) 5 years, (**c**) 15 years, and (**d**) 30 years.

**Figure 3:**
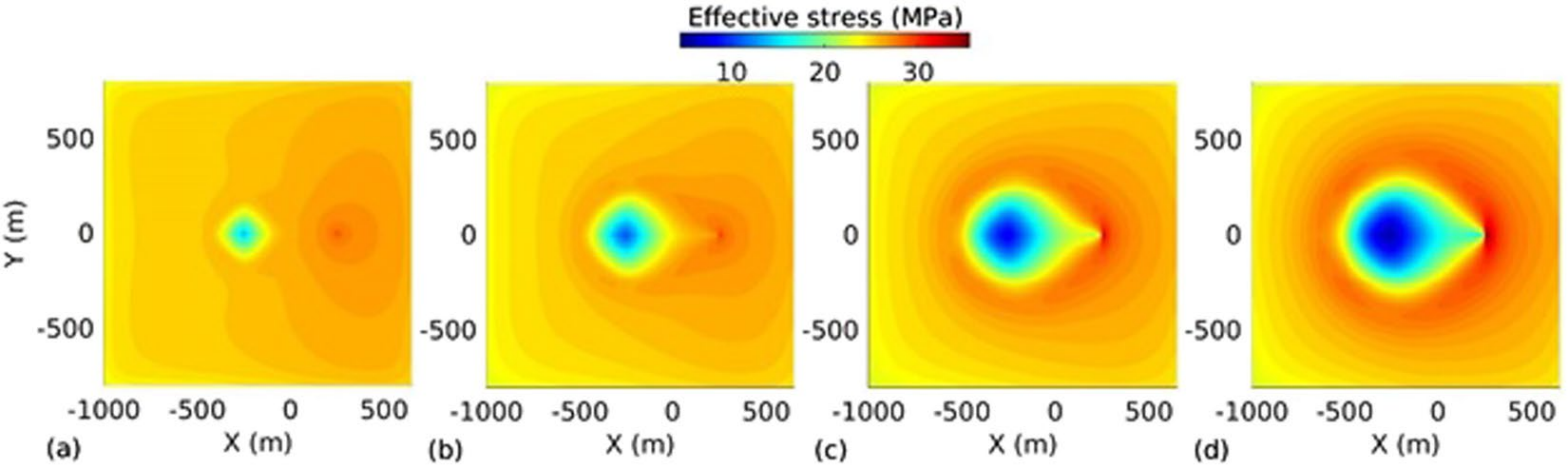
Effective stress distribution on the fracture plane at different time instances: (**a**) 1 year, (**b**) 5 years, (**c**) 15 years, and (**d**) 30 years.

**Figure 4:**
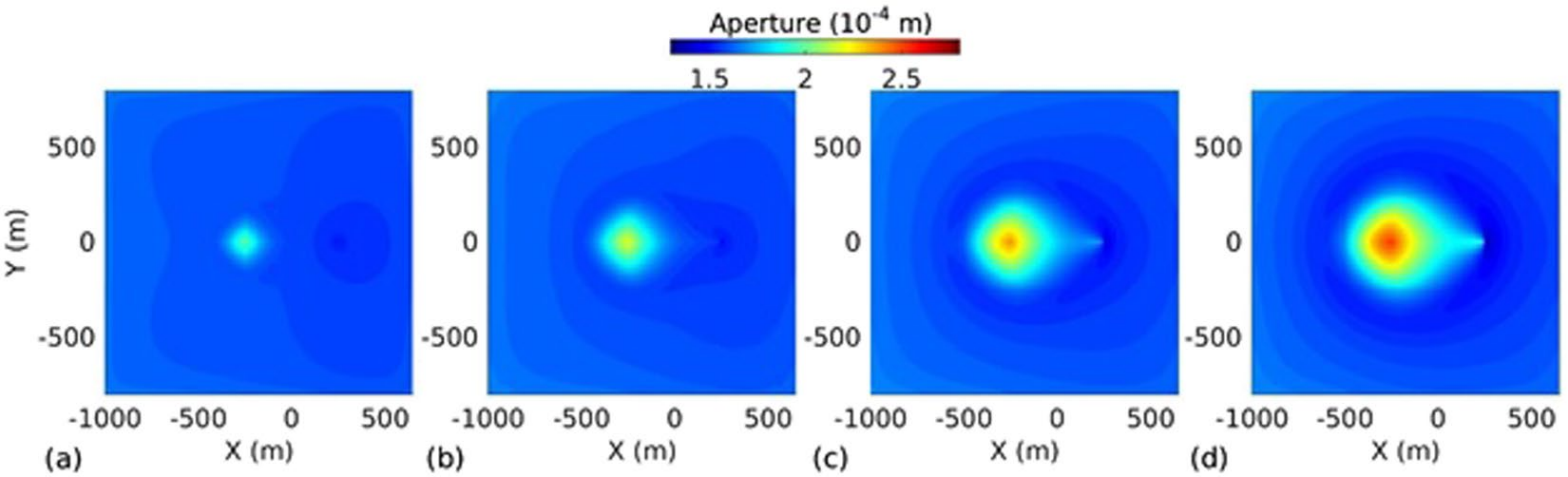
Aperture distribution on the fracture plane at different time instances: (**a**) 1 year, (**b**) 5 years, (**c**) 15 years, and (**d**) 30 years.

**Figure 5:**
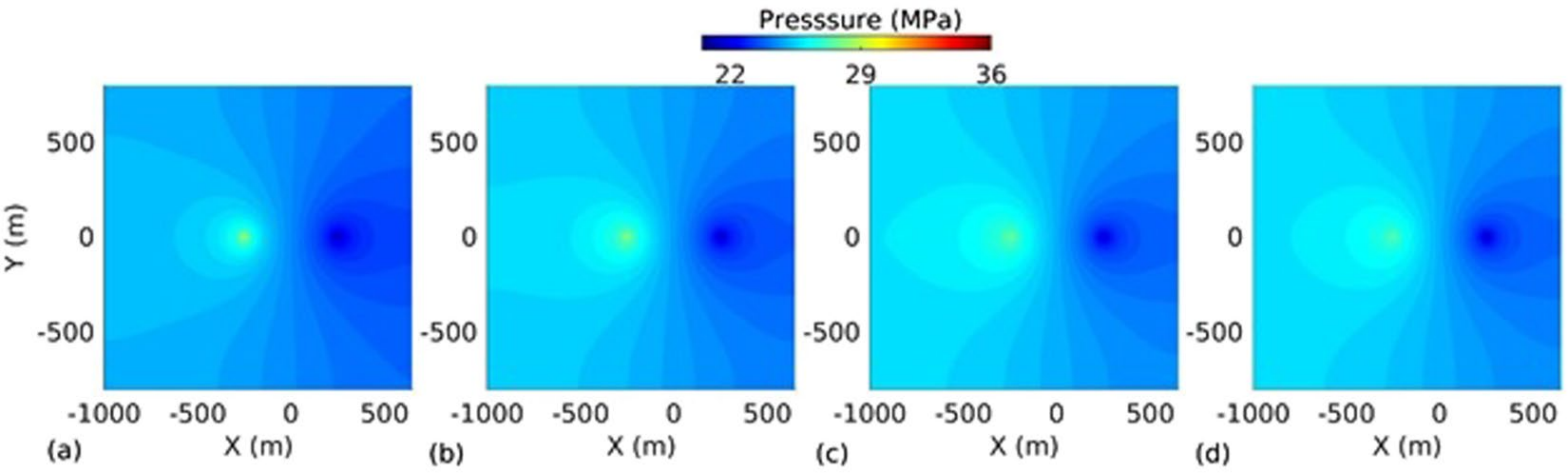
Fluid pressure distribution on the fracture plane at different time instances: (**a**) 1 year, (**b**) 5 years, (**c**) 15 years, and (**d**) 30 years.

**Figure 6:**
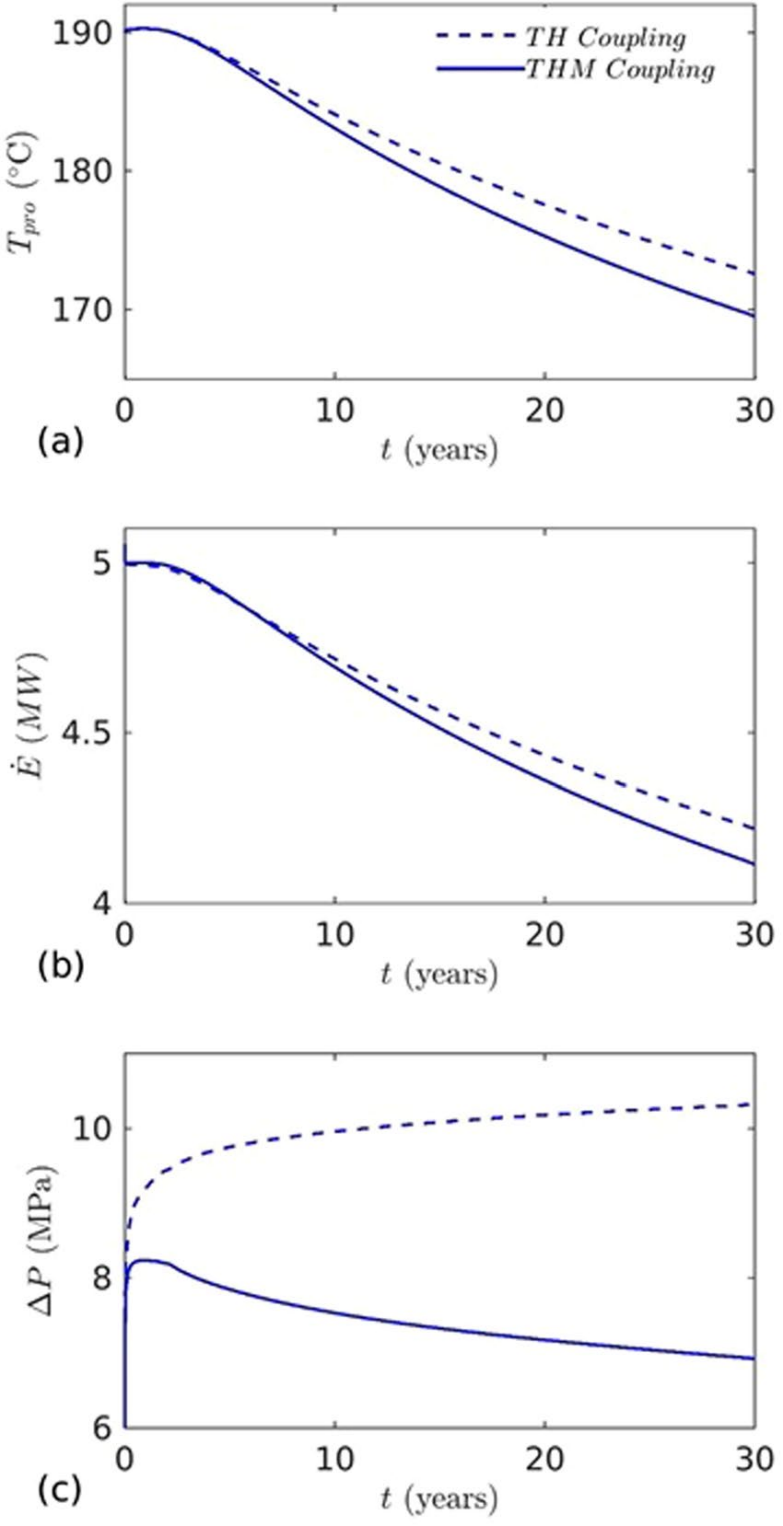
The effects of aperture alteration by thermal contraction and fluid overpressure are shown by comparing the results obtained from TH and THM simulations: (**a**) temperature at the production well, (**b**) heat extraction, and (**c**) pressure difference between injection and production wells.

Cold water injection into the fracture in an EGS reservoir increases the pore pressure and reduces the temperature of the reservoir. However, due to strong interactions among the flow, heat transfer and mechanical deformation, heat extraction from the reservoir is significantly influenced by the injection conditions and the coupling among the physical processes. The coupled effect of flow and induced deformation led to the spatio-temporal evolution of the fracture/reservoir. To investigate the effects of cold water injection on heat extraction performances and fracture/reservoir evolution due to cooling and pore pressure change, we conducted coupled thermo-hydro-mechanical (THM) simulations in the reservoir over a span of 30 years. The values of the reservoir and operational parameters are listed in Table 1. Prior to discussion of reservoir parameters and coupling effects in details, we discuss the base case of thermo-hydro-mechanical coupling. For the base case, we considered injection mass flow rate, *m* = 10 kg/s, and injection temperature, ${T}_{inj}\,=$ 70 °C, reservoir thermal gradients, *G* = 80 °C/km, reservoir permeability $k=1\times {10}^{-18}$ m^2^, and well distance, *W*_*L*_ = 500 m. The spatio-temporal evolution of temperature, effective stress, aperture and pressure on the fracture plane for the base case are presented in Figs 2, 3, 4 and 5 respectively. The temperature distribution on the fracture plane is plotted in Fig. 2 after 1, 5, 15 and 30 years. After 1 year (Fig. 2a) of operation, temperature drop occurred near to the injection well. The thermal front moves towards the production well, both cooling and thermal front movement on the fracture plane with time is clear from Fig. 2a–d. Temperature drawdown at production well with time is plotted in Fig. 6a. It was found that the cold front reached the production well after around 3.8 years and before that the production temperature is almost constant. Once the cold front reaches the production well, the temperature begins to drop at production. However, it is seen that the rate of decline of temperature at production well is faster with time after thermal breakthrough. This effect can be seen from the temperature-distance contour on the fracture plane (see Fig. 2). Figure 2a–d shows that the cooling areas do not increase with time as compared to the early days of injection/production. This is due to the opening of fracture in cooled zone, and closing outside where temperature drop is insignificant as shown in Fig. 4. This opening and closing of fracture tend to channelize the flow. It is hypothesized as the possible reason for faster temperature drop during later stages of injection/production. However, the fracture opening or closing is closely related to the evolution of effective stresses. The effective stress is altered by the combined effect of cooling and fluid pore pressure. Cooling induces tensile stress. The distribution of effective stress on fracture plane is shown in Fig. 3. It can be seen from Fig. 3a that the effective stress decreases more in the vicinity of the injection well, i.e. the zone that experiences more cooling. Meanwhile, the effective stresses increase towards and around the production well in early days as a result of the hot water travel. In early stage, the thermal front is also confined between the injection and production well (Fig. 2a). The combined poroelastic and thermoelastic effects tend to open the fracture around the injection well (Fig. 4a). However, the initial decrease in effective stress in the vicinity of injection well is controlled mainly by the poroelastic effects, whereas the thermal stresses play greater role in later periods of time. As cooling continues due to continuous injection of cold water, the effective stress field evolves with time (Fig. 3a–d). During middle to later stages of operation, a high permeable zone developed in mid-regions of the fracture (Fig. 4c,d) due to higher reduction in effective stresses in this zone by combined poro and thermoelastic effects. A high permeable channel also developed close to the production well despite less pronounced changes in effective stresses. This aperture increase may be a result of bending of fracture/reservoir due to opposite magnitude of stresses acting on fracture plane (tensile in cooled zone and compressive outside the cooled zone). Similar behavior was also reported in previous works^20,39^. With the formation of high permeable flow path, cold water gets channelized and causes faster temperature decline in the reservoir, including that at the production end. This effect becomes more significant after the thermal breakthrough. Figure 5 shows the spatio-temporal evolution of pressure on the fracture plane. In the early stage, fluid pressure near the injection well was high (Fig. 5a,b). Later the fluid pressure inside the fracture decreased with time (Fig. 5c,d) due to increase in fracture transmissivity.

**Table 1:** Input parameters^20,24,25^.

Parameter	Value
Fracture aperture (mm)	0.147
Reservoir rock permeability (m^2^)	1 × 10^−18^
Rock density (kg/m^3^)	2500
Water heat capacity (J/kg/K))	4180
Rock heat capacity (J/kg/K)	1000
Rock thermal conductivity (W/m/K)	2.9
Water thermal conductivity (W/m/K)	0.6
Young’s modulus of rock (GPa)	55
Poisson’s ratio of rock	0.30
Biot’s coefficient of rock	0.7
linear coefficient of thermal expansion (°C^−1^)	3 × 10^−5^
Joint stiffness (GPa/m)	100
Well spacing (m)	500, 550, 600 and 650
Injection rate (kg/s)	10, 15 and 20
Injection temperature (°C)	70 and 90

**Figure 7:**
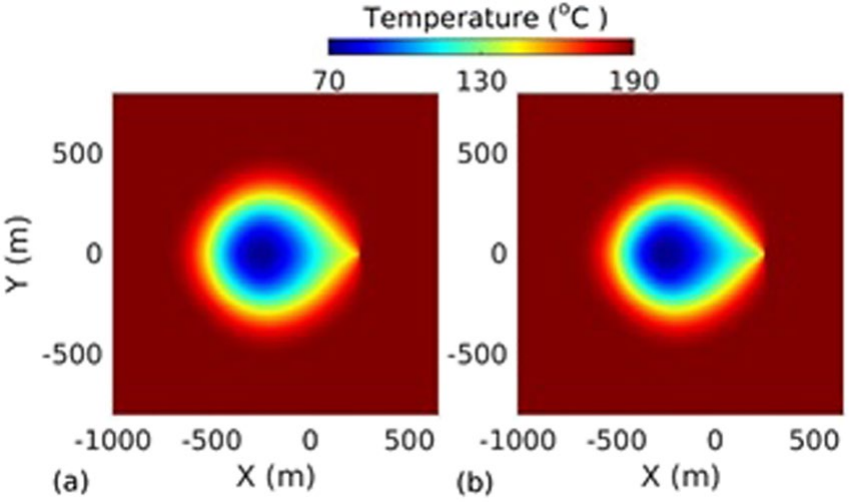
Temperature distribution on the fracture plane after 30 years (**a**) TH coupling and (**b**) THM coupling.

In order to quantify the coupling effects on heat extraction, we compared the results of thermo-hydro with the thermo-hydro-mechanical process. In thermo-hydro processes, the flow induced deformation (poroelastics and thermoelastics effect) is ignored, resulting in larger flow path and slower temperature drop at the production well. The temperature distributions on fracture plane after 30 years for TH and THM are shown in Fig. 7a and b. From Fig. 7a, it can be seen that the thermal affected zone is larger and cooling areas are uniform than the THM case as shown in Fig. 7b. However, due to larger cooling area, injected water extracts more heat from the reservoir before reaching to production well (Fig. 6b). In TH process, pressure difference with time increases due to decrease of effective transmissivity. The effective transmissivity of fracture is decreased by cooling which causes more viscous resistance. This effect is clearly visible in Fig. 6c and plotted by the dotted line. The relative pressure difference of TH and THM processes increased with time (Fig. 6c). In THM process, pressure decrease with time was a result of fracture opening that enhanced fracture transmissivity (Fig. 4). This reduced the pressure difference to circulate the fixed mass of injected water. However, in early stage (~1 year), slight increase of pressure was observed (Fig. 6c). This was the result of minor fracture closure ahead of the production well (Fig. 4a). Figure 6c shows that the pressure difference at the end of production is ~32% higher than the THM case. The pressure effect on heat extraction is very minor and it is ~2.46% higher than THM case after 30 years. However, in TH case, heat extraction rate from the reservoir is slightly less up to ~5.8 years due to high injection pressure. The quantitative comparison of performance of both processes after 30 years are presented in Table 2. The results of this study confirm that the thermal and mechanical stresses play a very important role in energy production from the reservoir and are coupled to various parameters and factors. Ignoring their effects may lead to remarkable under/overestimation of energy production from the geothermal reservoir.

**Table 2:** *m* = 10 kg/s, *T*_*inj*_ = 70 °C, *W*_*L*_ = 500 m and *G* = 80 °C/km.

Parameter	TH Process	THM Process	% change from TH to THM
Temperature (°C)	172.518 (life time ~ 30.8 Years)	169.52 (life time ~ 29.22 Years)	1.7378%
Energy (MW)	4.2184	4.1145	2.4630%
Pressure (MPa)	10.315	6.925	32.86%

**Figure 8:**
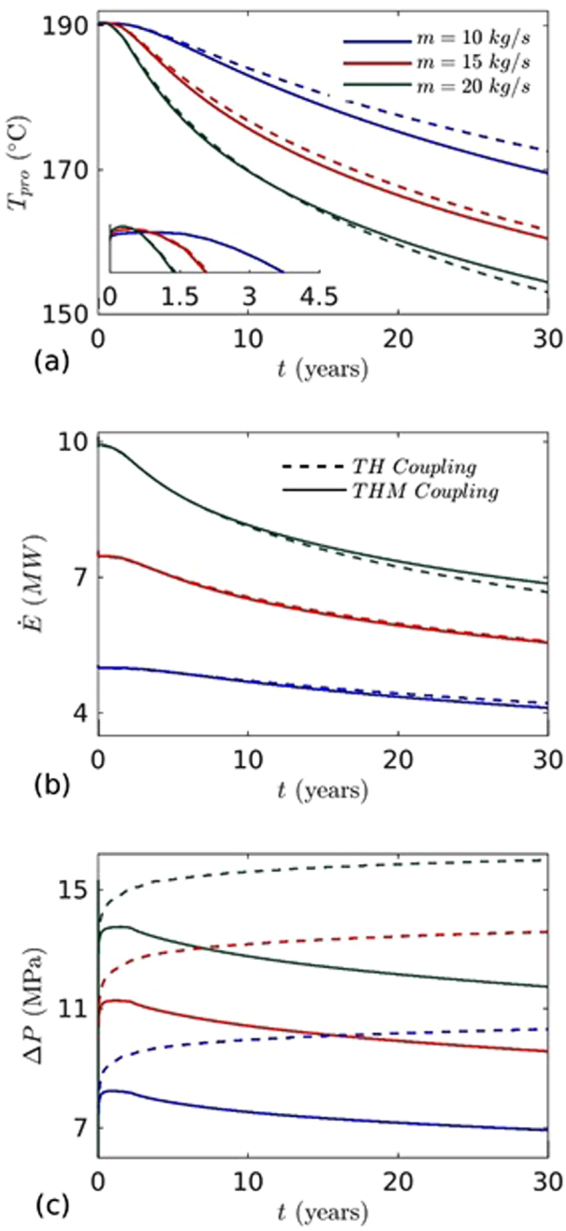
The effects of mass flow rates on: (**a**) temperature at the production well, (**b**) heat extraction, and (**c**) pressure difference between injection and production wells. Injection temperature (*T*_*inj*_ = 70 °C) and well distance (*W*_*L*_ = 500 m) are fixed.

To further confirm these effects, we performed an independent sensitivity analysis of the important parameters. The effects of injection mass flow rate, temperature, well distance, reservoir permeability and geothermal gradient on energy production were investigated. To investigate the effects of mass flow rate and injection temperature, three different values of mass flow rate (10, 15 and 20 kg/s) and two values of fixed injection temperature (70 and 90 °C) are considered. Figure 8 shows the variation of temperature, pressure difference and heat extraction rate at production well with time for 70 °C injection temperature. It was found that with mass flow rate increment from 10 to 20 kg/s with a step increase of 5 kg/s, the heat extraction rate from the reservoir increased significantly, but led to relatively early thermal breakthrough at production well. The advection inside the fracture increased with the mass flow increase and water travelled longer distance inside the fracture. However, after thermal breakthrough the heat extraction rate and temperature drops were relatively faster. This is due to slow heat conduction in low permeable surrounding rock matrix. The direct relation of mass flow rate on heat extraction is evident from eq. (11). Unpredicted behavior is observed in TH and THM case for higher mass of injection (*m* = 20 kg/s). In the latter case, we found almost same temperature drop at the production well up to ~14 years (Fig. 8a). The results show that coupling effects on reservoir evolution due to THM is almost negligible on temperature drop at production well. However, after around ~14 years, the temperature drop at production well was higher for TH case (plotted in Fig. 8a by dotted green line). On the other hand, the coupling effect is slowly decreasing with increasing the mass flow rate. It is concluded that the mass flow rate and the coupling effects play a dominating role in the thermal profile evolution of a reservoir and optimum flow rate must be determined prior to initiating extraction of heat from an EGS.

**Figure 9:**
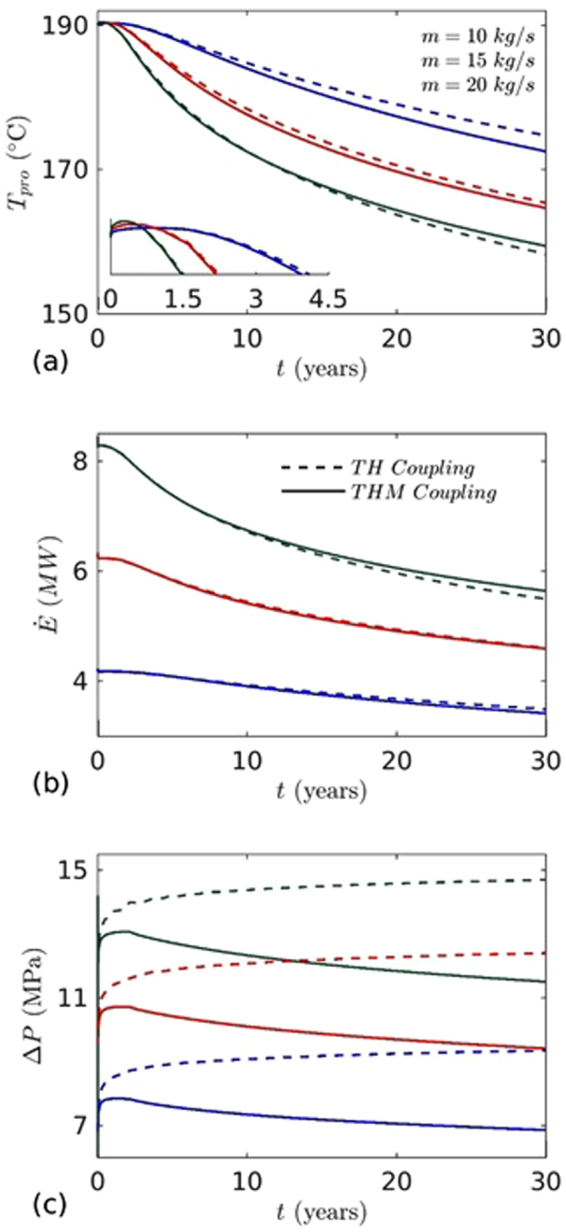
The effects of mass flow rates on: (**a**) temperature at the production well, (**b**) heat extraction, and (**c**) pressure difference between injection and production wells. Injection temperature (*T*_*inj*_ = 90 °C) and well distance (*W*_*L*_ = 500 m) are fixed.

We further performed the simulations and analysis for higher injection temperature (*T*_*inj*_ = 90 °C). The results showed almost similar behavior (Fig. 9a). However, the heat extraction rate decreased significantly for all mass flow rates. The heat extraction decline at higher injection temperature (*T*_*inj*_ = 90 °C, as compared to 70 °C) of TH and THM cases for mass flow rate 10, 15 and 20 kg/s are 17.20, 17.53, 17.63 and 16.94, 17.39, 17.78% (Tables 3 and 4). This is due to the lower temperature difference between injected water and the fracture/reservoir temperature. In this case the reservoir cools slowly. Figures 8c and 9c show the pressure difference between the injection and production wells for two cases of injection temperatures. The pressure difference increased with the increase in the mass flow rate while it decreased with the increase in injection temperatures. This means that a higher injection temperature case would require less pumping power (${P}_{E}=m{\rm{\Delta }}P/\eta \rho$) than the lower injection temperature case. The pressure difference decreases faster in lower injection temperature case. This indicates that the permeability evolution due to thermal stress is more. Past study suggests that the energy extraction becomes less economical when the temperature of production well declines by 10%^40^. Based on that, the life time of higher mass injection case (*m* = 20 kg/s,) in TH and THM coupling process is 20.53 and 21.26 years (Table 5). However, it is around 30.80 and 29.22 years for *m* = 10 kg/s (Table 2). These results highlight an important relationship between the injection mass flow rate and life time of the reservoir and should be a key consideration for any EGS operation

**Table 3:** *m* = 10 kg/s, *T*_*inj*_ = 90 °C, *W*_*L*_ = 500 m and *G* = 80 °C/km.

Parameter	TH Process	THM Process	% change from TH to THM
Temperature (°C)	174.7 (life time ~ 31.94 Years)	172.50 (life time ~ 30.78 Years)	1.2593%
Energy (MW)	3.4926	3.4173	2.1560%
Pressure (MPa)	9.354	6.858	26.6838%

**Table 4:** T_*inj*_ = 90 °C, *W*_*L*_ = 500 m and *G* = 80 °C/km.

Parameter	TH (m = 15 kg/s)	THM (m = 15 kg/s)	TH (m = 20 kg/s)	THM (m = 20 kg/s)
Temperature (°C)	165.37 (life time ~ 27.03 Years)	164.65 (life time ~ 26.65 Years)	158.28 (life time 23.3 Years)	159.38 (life time ~ 23.88 Years)
Energy (MW)	4.6023	4.5910	5.4938	5.6390
Pressure (MPa)	12.406	9.413	14.7	11.506

**Table 5:** T_*inj*_ = 70 °C, *W*_*L*_ = 500 m and *G* = 80 °C/km.

Parameter	TH (m = 15 kg/s)	THM (m = 15 kg/s)	TH (m = 20 kg/s)	THM (m = 20 kg/s)
Temperature (°C)	161.59 (life time ~ 25 Years)	160.45 (life time ~ 24.44 Years)	153.02 (life time ~ 20.53 Years)	154.41 (life time ~ 21.26 Years)
Energy (MW)	5.5807	5.5575	6.6703	6.8589
Pressure (MPa)	13.586	9.571	15.997	11.739

**Figure 10:**
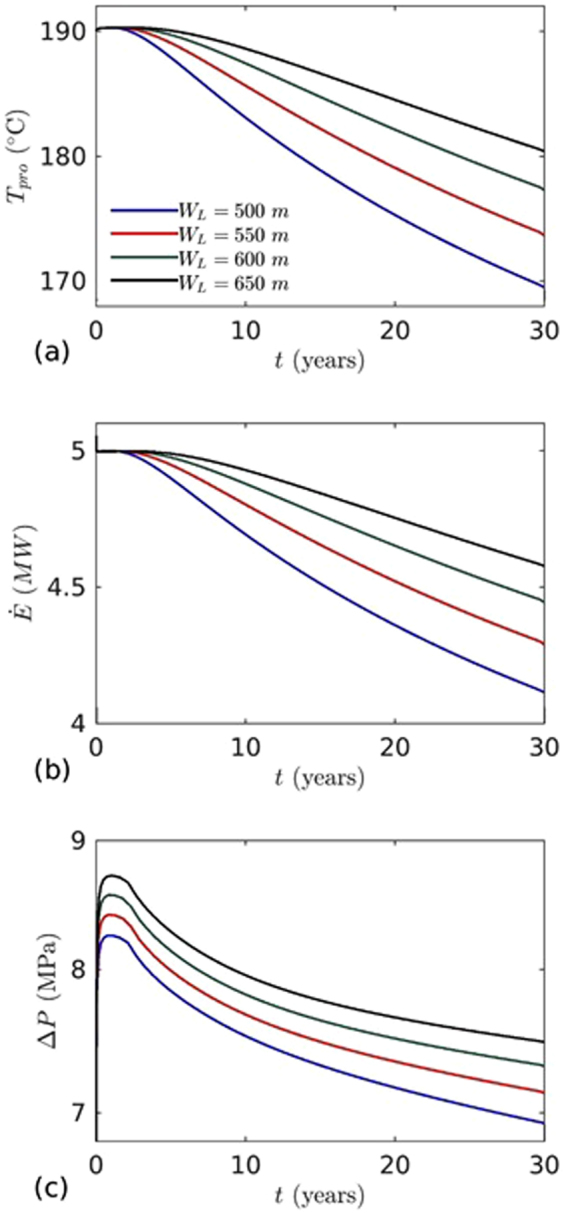
The effects of well distance on: (**a**) temperature at the production well, (**b**) heat extraction, and (**c**) pressure difference between injection and production wells. Injection mass flow rate (*m*_*inj*_ = 10 kg/s) and temperature (*T*_*inj*_ = 70 °C) are fixed.

Figure 10 shows the effects of well distance on ${T}_{inj}\,,\,\dot{E}$ and ${\rm{\Delta }}P$. Four well distances, 500, 550, 600 and 650 m were considered. The thermal breakthrough at production well delayed with increase in well distance; for instance after 30 years, the temperatures at production well were found to be 2.37, 4.37 and 6.03% higher than the base case (*W*_*L*_ = 500 m) as presented in Table 6. While increasing well distance significantly increased the heat extraction from the reservoir, these would increase the pressure differences and consequently, lead to higher pumping power.

**Table 6:** *m* = 10 kg/s,*T*_*inj*_ = 70 °C and *G* = 80 °C/km (THM Process).

Parameter	550 m	% change from 500 to 550	600 m	% change from 500 to 600	650 m	% change from 500 to 650
Temperature (°C)	173.646 (life time ~ 31.4 Years)	2.3761%	177.277 (life time ~ 33.3 Years)	4.3756%	180.396 (life time ~ 34.94 Years)	6.0290%
Energy (MW)	4.2897	4.0842%	4.4443	7.4207%	4.5774	10.1127
Pressure (MPa)	7.137	2.9704%	7.324	5.4478%	7.493	7.5804%

**Figure 11:**
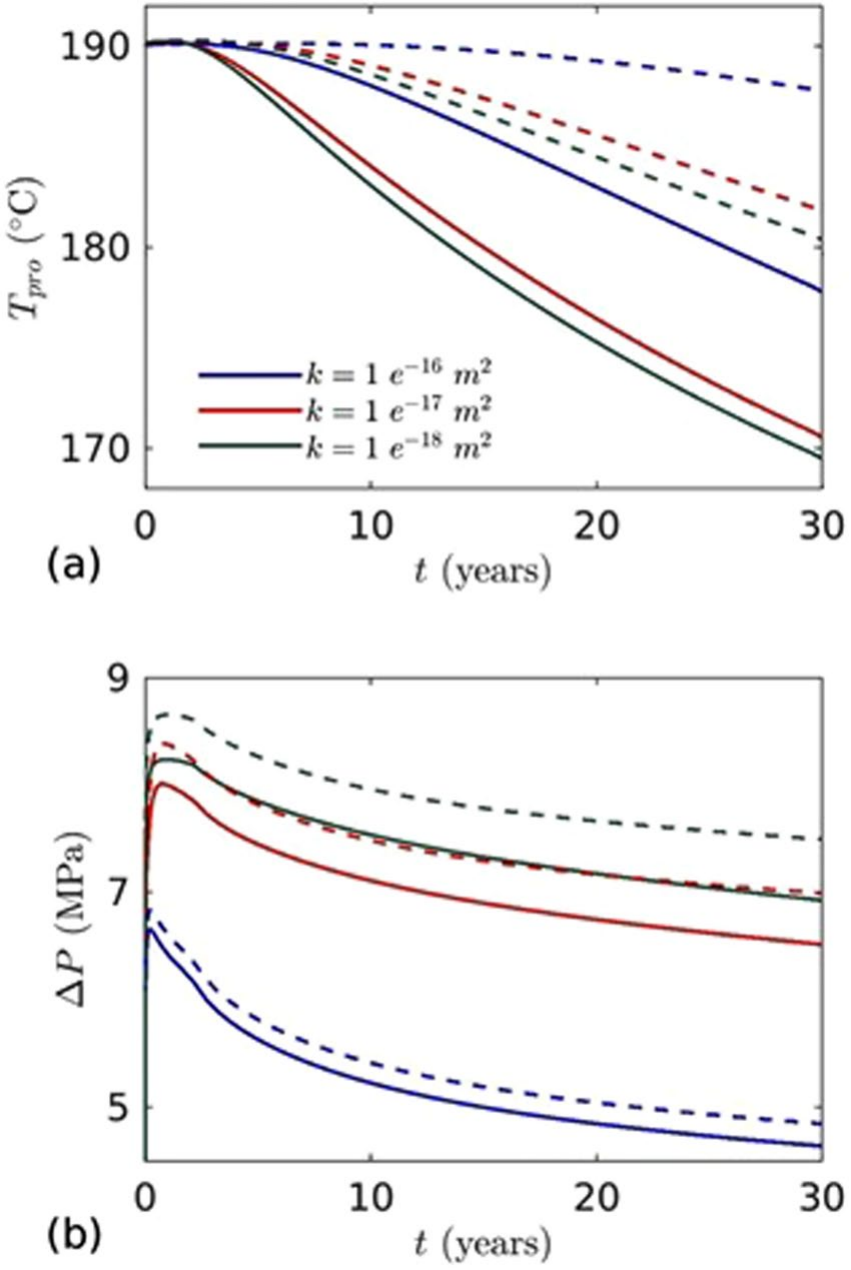
The effects of reservoir permeability on: (**a**) temperature at the production well, and (**b**) pressure difference between injection and production wells. Solid line for well distance 500 m and dotted line for well distance 650 m. Injection mass flow rate (*m*_*inj*_ = 10 kg/s) and temperature (*T*_*inj*_ = 70 °C) are fixed.

Another important parameter for an EGS is reservoir permeability. A high permeability of the rock matrix will decrease the amount of water reaching at the production well. In fact, water loss into the reservoir will add an extra cost of energy production and reduce the heat extraction from the reservoir. To investigate the effects of reservoir permeability, coupled THM simulations were performed for different values of permeability, $k=1\times {10}^{-16}$, $k=1\times {10}^{-17}$ and $k=1\times {10}^{-18}$ m^2^. The simulations were performed for the base case and for well length, ${W}_{L}=$ 650 m. The results presented in Fig. 11 show the permeability and well distance effect on ${T}_{inj}\,$ and ${\rm{\Delta }}P$. Figure 11a shows that on increasing the reservoir permeability from $k=1\times {10}^{-18}$ to $k=1\times {10}^{-16}$, the temperature at the production well decreased slowly and delayed the thermal breakthrough time. This indicates that less amount of water reaches the production well. This effect can be seen from the ${\rm{\Delta }}P$ vs. $t$ plot in Fig. 11b. It shows that the pressure difference decreased significantly for higher reservoir permeability. Similarly, with increasing well distance, the temperature at production well was higher because larger amount of cold water was lost into the reservoir matrix. The comparative effects can be noticed in Figs 10c and 11b.

**Figure 12:**
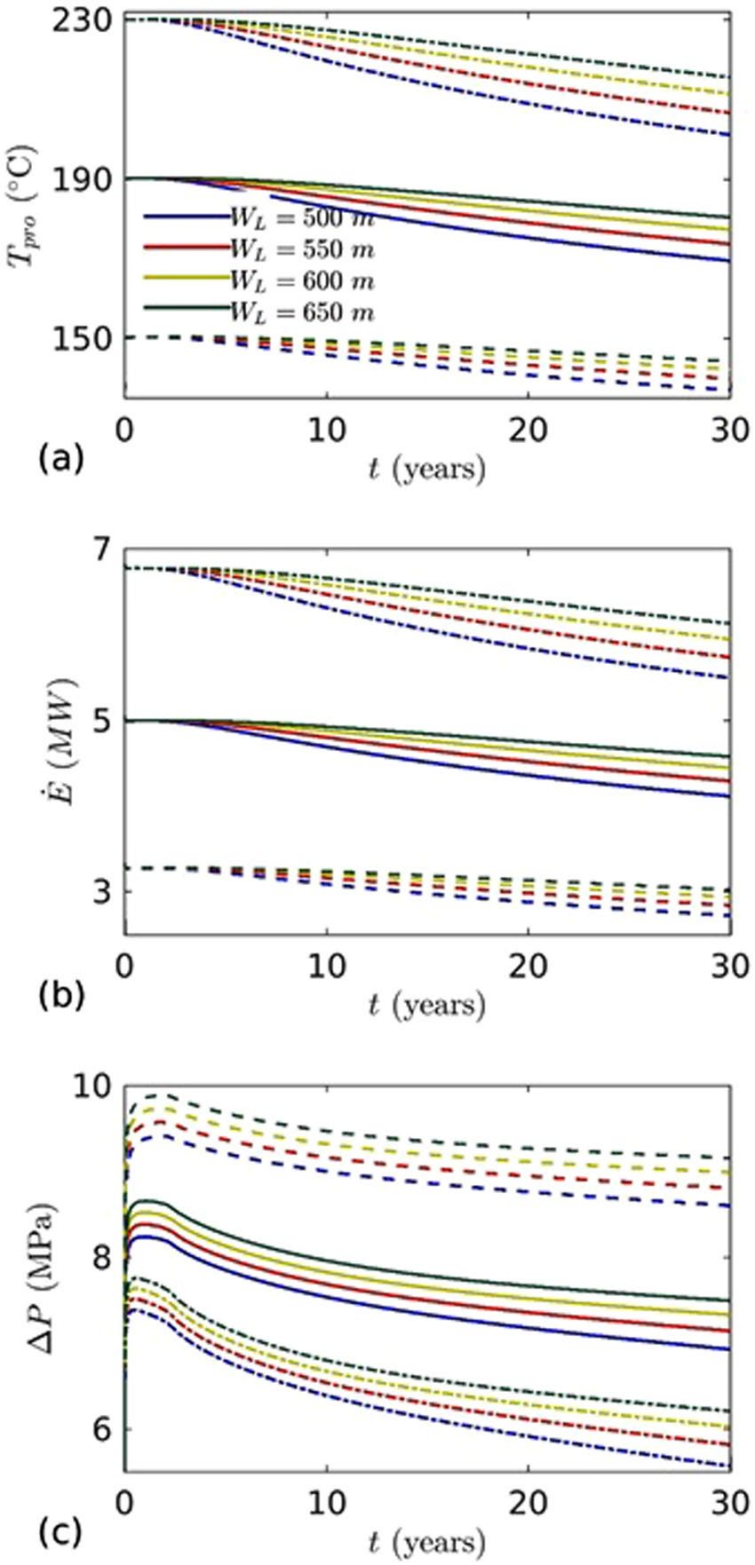
The effects of geothermal gradient and well distance on: (**a**) temperature at the production well, (**b**) heat extraction, and (**c**) pressure difference between injection and production wells. Dotted line for geothermal gradient 60 °C/km, solid line for geothermal gradient 80 °C/km, and dotted-dashed line for geothermal gradient 100 °C/km. Injection mass flow rate (*m*_*inj*_ = 10 kg/s) and temperature (*T*_*inj*_ = 70 °C) are fixed.

Figure 12 shows ${T}_{inj}\,,\,\dot{E}$ and ${\rm{\Delta }}P$ vs. $t$ curve for three geothermal gradients, *G* = 60, 80 and 100 °C/km. The temperatures of fracture at a depth of 2 km are 150, 190 and 230 °C. The temperature and mass flow rate of injected water are 70 °C and 10 kg/s. The reservoir rock properties were considered same as the base case. It was found that temperature drop at production well was strongly influenced by geothermal gradient (Fig. 12a). For higher geothermal gradient (*G* = 100 °C/km), the temperature drop at production well were much higher and more affected by the well distance. As expected, the thermal stress effects are higher, resulting in faster evolution of fracture transmissivity. However, heat extraction rate is higher but the ${T}_{inj}/\dot{E}/{\rm{\Delta }}P$ drops are relatively faster than the lower geothermal gradients with time (Fig. 12b). For lower geothermal gradients, the pressure difference decreases slowly when compared with higher geothermal gradient (Fig. 12c). In this case, the fracture transmissivity evolution is reduced due to the thermal effects. Higher pressures are required to circulate the water for lower geothermal gradients (Fig. 12c).

## Conclusion

In this work, coupled thermo-hydro-mechanical and thermo-hydro simulations of a 3D enhanced geothermal system were studied. The effects of coupling processes, operating parameters and reservoir rock formation on heat extraction were investigated. The results show that effective stresses decrease due to cooling and fluid overpressure, that leads to the opening of fracture aperture. The effective stresses decrease more near the injection well as the fluid pressure is relatively high and the rock gets cooled very fast in this zone. With the combined effects of poro- and thermo-elastic stresses, the aperture grows ~2 times of its initial value. The aperture growth at early days is controlled by the poroelastics stress. The thermoelastics stress became dominant at later stages. However, the effective stress increased in a zone outside of cooling region and beyond the production well leading to fracture closure. The fracture aperture closing and opening creates the high permeable zone between the wells, and the injected water get channelized and reach the production well quickly. Opening of the fracture reduced the injection pressure needed to circulate the water. For lower injection mass flow rate, *m* = 10 kg/s, the temperature drop at the production well was slower for TH coupling than THM coupling. The differences start reducing with increase in the mass flow rate. At higher injection mass, *m* = 20 kg/s, we found almost same temperature at production well for up to 14 years of operation in both the coupled cases. After that the temperature at production well in TH coupling dropped to a lower value than that in the THM coupling. This finding contradicts the earlier studies that showed that transmissivity evolution creates channelized flow. This does stand true in our study but only in lower injection mass cases. However, slower drops of temperature in THM coupling for higher injection mass, *m* = 20 kg/s, may be a result of higher flow paths. In case of the latter, the poroelastics effect is higher resulting in the higher increase in fracture permeability around the injection side despite the same thermal stresses. Similar trends are also noticed in higher injection temperature (${T}_{inj}\,=$ 90 °C). This indicates that coupling of these processes depend on various factors and they need to be evaluated in each investigation. It is worthy to mention here that the behavior cannot be highlighted without considering a large domain equal to a typical size of an EGS reservoir. We conclude that simplified TH coupling may be sufficient to predict the behavior of an EGS reservoir, if all the parameters and coupling among the processes are suitably accounted for.

On studying the role of inter-well spacing, we found that the heat extraction performances increased with increasing the well lengths. At higher well spacing, the flow length/volume increased and so did the pumping power, leading to improved overall performance in heat extraction. The results showed that water loss inside the reservoir increased with increasing reservoir permeability. Further, the geothermal gradients significantly influenced the heat extraction performances. More heat extraction occurred in higher geothermal gradients, but steep decrease of ${T}_{inj}\,,\,\dot{E}$ and ${\rm{\Delta }}P$ occurred with time. The present developed geothermal model in THM framework can improve the understanding of fracture dominated EGS reservoirs. The modeling results can also be useful in predicting the life time based on the operating conditions.
